# GC-TOF-MS-Based Metabolomics Analyses of Liver and Intestinal Contents in the Overfed vs. Normally-Fed Geese

**DOI:** 10.3390/ani10122375

**Published:** 2020-12-11

**Authors:** Minmeng Zhao, Ya Xing, Lidong Liu, Xiang Fan, Long Liu, Tuoyu Geng, Daoqing Gong

**Affiliations:** 1 College of Animal Science and Technology, Yangzhou University, Yangzhou 225009, China; mmzhao@yzu.edu.cn (M.Z.); xingya325@163.com (Y.X.); ld.lau3698@hotmail.com (L.L.); fan-x19@foxmail.com (X.F.); liujiaolong688@sina.com (L.L.); 2 Joint International Research Laboratory of Agriculture and Agri-Product Safety of the Ministry of Education of China, Yangzhou University, Yangzhou 225009, China

**Keywords:** non-alcoholic fatty liver disease, metabolomics, intestinal tracts, goose

## Abstract

**Simple Summary:**

Non-alcoholic fatty liver disease has been considered as one of the most important causes of liver disease, and it is a threat to human and animal health worldwide. Interestingly, goose fatty liver can reach 8–10 times the weight of normal liver with no overt pathological symptoms, suggesting that there are some protective mechanisms. Scientists have indicated that gut microbiota participate in the formation of non-alcoholic fatty liver disease in human and mammalian animals. However, it is unclear whether gut microbiota and their metabolites contribute to goose fatty liver. The aim of the present study was to investigate the metabolomic analyses of liver and intestinal contents in overfed vs. normally fed geese. The results showed that the formation of goose fatty liver is accompanied by obvious changes in the metabolic profiles of liver and intestinal contents. The intestinal metabolites can affect the formation of goose fatty liver by affecting the metabolisms of glucose and fatty acid, oxidative stress, and inflammatory reactions. These findings provide a basis for future work addressing the relationship between intestinal metabolites and the development of non-alcoholic fatty liver disease.

**Abstract:**

No overt pathological symptoms are observed in the goose liver with severe steatosis, suggesting that geese may host unique protective mechanisms. Gas chromatography time-of-flight mass spectrometry-based metabolomics analyses of liver and intestinal contents in overfed vs. normally fed geese (26 geese in each treatment) were investigated. We found that overfeeding significantly changed the metabolic profiles of liver and intestinal contents. The differential metabolites mainly belong to fatty acids, amino acids, organic acids, and amines. The differential metabolites were involved in glycolysis/gluconeogenesis, glycerolipid metabolism, the pentose phosphate pathway, fatty acid degradation, the sphingolipid signaling pathway, and the biosynthesis of unsaturated fatty acids. Moreover, we determined the biological effects of arachidonic acid (ARA) and tetrahydrocorticosterone (TD) in goose primary hepatocytes and intestinal cells. Data showed that the mRNA expression of arachidonate 5-lipoxygenase (*ALOX5*) in goose primary intestinal cells was significantly induced by 0.50 mM ARA treatment. Cytochrome P-450 27A1 (*CYP27A1*) mRNA expression was significantly inhibited in goose primary hepatocytes by 1 µM TD treatment. In conclusion, the formation of goose fatty liver is accompanied by significant changes in the metabolic profiles of liver and intestinal contents, and the changes are closely related to the metabolisms of glucose and fatty acids, oxidative stress, and inflammatory reactions.

## 1. Introduction

Non-alcoholic fatty liver disease (NAFLD) in humans and mammals generally contains a disease spectrum ranging from simple steatosis to non-alcoholic steatohepatitis (NASH), fibrosis, cirrhosis, and ultimately liver cancer [1]. Previous studies have demonstrated that genetic factors, abnormal lipid metabolism, insulin resistance, oxidative stress, and inflammation contribute to the development of NAFLD [2,3]. The intestinal microflora can affect digestion and the absorption and metabolism of carbohydrates, proteins, and fats, thus regulating the nutrition level, health status, and immunity of the host. Therefore, intestinal microflora play an important role in nutrient metabolism, intestinal development, immunity, and the occurrence of various diseases in animals [4]. Many studies have indicated that gut microbiota participate in the formation of NAFLD, obesity, and disorders associated with lipid metabolism, and one of the mechanisms is mediated by the metabolites produced by intestinal bacteria [5,6].

Goose fatty liver can reach 8–10 times the weight of normal liver after approximately twenty days of overfeeding, and the fat level is over 60% [7]. Interestingly, no obvious injury or pathological symptoms such as inflammation or hepatic fibrosis are found, suggesting that there are some protective mechanisms in the formation of goose fatty liver [8,9]. Our previous study indicated that lactic acid from the gut microbiota is associated with the suppression of inflammation, implying that gut microbiota are a component of the protective mechanism preventing the progression from simple steatosis to NASH in goose liver [10]. However, it remains unclear whether the formation of goose fatty liver is accompanied by changes in the metabolic profiles of liver and intestinal contents and whether metabolites generated by gut microbiota contribute to the formation of goose fatty liver. Therefore, the objective of the present study was mainly focused on performing metabolomics analyses of liver and intestinal contents in overfed vs. normally fed geese. The results may not only promote the understanding of the mechanisms underlying the formation of goose fatty liver but also provide a new solution to NAFLD-associated problems in humans and other economic animals.

## 2. Materials and Methods

### 2.1. Animal Experiment

All animal protocols were approved by the Yangzhou University Animal Ethics Committee (NSFC2020-DKXY-22, 23 March 2020). A total of 52 healthy 70-day-old male Landes geese with similar body weight were purchased from Li Cheng Farm (Huai’an, China). These geese were randomly divided into a control group (fed with free access to feed and water) and a treatment group (overfed) (26 geese each group). The geese were raised in cages with two geese per cage (length 70 cm, width 60 cm, height 60 cm) in a temperature-controlled room (the temperature was set at 23 to 25 °C and relative humidity was kept at 60% to 65%). The diet contained maize boiled for 5 min plus 1% plant oil, 1% salt, and 0.2% multiple vitamins). In the treatment group, the geese were overfed using the method described previously [11]. Briefly, geese in the treatment group were subjected to 7 days of pre-overfeeding and the daily feed intake was gradually increased from 100 to 300 g. This was followed by a formal overfeeding program for 24 days, which was as follows: 500 g for 5 days (three meals a day), 800 g for one week (four meals a day), followed by 1200 g in the remaining time (five meals a day). Twelve geese from each treatment were randomly selected and killed by cervical dislocation on 82 and 94 days of age. The liver samples and intestinal content samples from jejunum, ileum, and cecum were collected and stored at –80 °C.

### 2.2. Treatment of Goose Primary Cells with Metabolites

The primary hepatocytes and intestinal cells were isolated from 23-day-old Landes goose embryos. In brief, the liver and intestinal tract were harvested from the embryos, followed by digestion with 0.1% type IV collagenase (Worthington Biochemical Corporation, Lake Wood, NJ, USA) at 37 °C for 25 min. Complete culture medium was prepared with the mixture of high-glucose Dulbecco’s modified eagle medium (DMEM, Gibco, USA), 10% fetal bovine serum (Gibco, USA), 100 IU/mL penicillin (Sigma-Aldrich, St. Louis, MI, USA), 100 mg/mL streptomycin (Sigma-Aldrich, St. Louis, MI, USA), and 0.02 mL/L epidermal growth factor (Peprotech, UK), added to terminate the digestion. Subsequently, the mixture was filtered through a 0.22 μm filtration membrane to obtain the cell suspension. After the suspension was centrifuged at 700× *g* for 8 min, the red blood cell lysis buffer (Solarbio Co., Ltd., Beijing, China) was added to the cell pellet for 12 min. Centrifugation was again performed at 700× *g* for 12 min to spin down the cells. After the cells were rinsed with complete culture medium twice, the cell pellet was re-suspended in complete culture medium. Lastly, the cells were counted, diluted with complete culture medium to 1 × 10^6^ cells/mL, and transferred to 5% CO_2_ incubator at 38 °C for cell culture.

After 24 h of incubation, the cells were rinsed with phosphate-buffered saline twice after the culture medium was removed. For arachidonic acid (ARA) treatment, hepatocytes and intestinal cells were treated with 0.25 mM or 0.5 mM ARA for 14 h, and those treated with complete culture media only were considered as the control. The ARA solution preparation and treatment of cells was conducted according to the method reported by Zhang et al. [12]. In brief, 0.125 mmol of ARA (Sigma, St. Louis, MO, USA) was dissolved in 0.5 mL of NaOH (0.5 M) at 70 °C and then mixed with 2 mL of 10% bovine serum albumin (BSA). Afterwards, the mixture was diluted in DMEM at 37 °C to obtain 50 mM stock solution. For tetrahydrocorticosterone (TD) treatment, a final concentration of 0.2 μM or 1 μM TD conjugated with BSA was made in complete culture medium. Hepatocytes and intestinal cells treated with complete culture media plus 2% BSA and 0.2% alcohol were used as the controls. There were six replicates for each treatment. After treating cells for 14 h, the cells were rinsed with PBS, followed by harvesting with TRIzol reagent (Invitrogen, Carlsbad, CA, USA).

### 2.3. Sample Preparation for Metabolomics Analysis

Samples from liver and intestinal content of jejunum, ileum, and cecum were weighed and transferred into 2 mL tubes and mixed with 450 μL of extracting solution (methanol and chloroform, 3:1, *v*/*v*) and 10 μL of 2-Chloro-L-phenylalanine and vortexed for 30 s. Subsequently, all samples were homogenized using a grinding mill (Jingxin Industrial Development Co., LTD, Shanghai, China) for 240 s at 45 Hz and treated in ultrasonic apparatus (Radbon Electronics Co. LTD, Shenzhen, China) for 300 s while being incubated in ice water. After the mixture was centrifuged at 12,000× *g* for 15 min at 4 °C, the supernatant (350 μL) was carefully transferred into a 2 mL silylated sample injector and desiccated in a vacuum freeze drier (Huamei Biochemical instrument Factory, Taicang, China). Meanwhile, 50 μL of supernatant from each sample was mixed as a quality control (QC) sample. The dried samples were reacted with 50 μL of methoxy aminatio hydrochloride (dissolved in pyridine) for 30 min at 80 °C, followed by the addition of 70 μL of bis-(trimethylsilyl)-trifluoroacetamide reagent (including 1% trimethylchlorosilane), and incubated for 90 min at 70 °C. Afterwards, 5 μL of fatty acid methyl esters (dissolved in chloroform) was added when the mixture was cooled to room temperature.

### 2.4. GC-TOF-MS Analysis

Based on the methods of Yang et al. [13] and Wen et al. [14], the samples were analyzed using an Agilent 7890 gas chromatograph (Agilent Corporation, Santa Clara, CA, USA) coupled with a time-of-flight mass spectrometry (GC-TOF-MS) system, and a DB-5MS capillary column (30 m × 0.25 mm × 0.25 μm, J & W Scientific, Folsom, CA, USA) was selected to separate the derivatives. The sample volume was 1 μL by splitless mode. Column flow was set at 1 mL/min, and helium was used as the carrier gas. The oven temperature programs were set up as follows: 50 °C hold on for 1 min, then raised to 310 °C at a rate of 10 °C/min, and maintained for 8 min. In addition, the temperatures for front injection, transfer line, and ion source were 280, 280, and 250 °C, respectively. Electron energy of 70 eV and solvent delay time of 6.27 min was applied. The QC sample was analyzed at regular intervals (every twelve samples) to monitor the stability of the instrument. In addition, a blank sample (the vehicle acetonitrile) was used to rinse the column every three samples so that there was no residual sample left in the column.

### 2.5. Real-Time PCR Analysis

Total RNA was extracted from cell samples according to the instructions of the Trizol Reagent manufacturer (TaKaRa Biotechnology Co. Ltd., Dalian, China). The total RNA level and quality was evaluated by a spectrophotometer (Nano Drop Technologies, Inc., Wilmington, DE, USA). Total RNA was reverse-transcribed into cDNA with commercial kits (TaKaRa Biotechnology Co. Ltd., Dalian, China). Real-time PCR was carried out on an ABI 7500 RT-qPCR System (Applied Biosystems, Foster City, CA, USA). The cycling conditions were: 95 °C for 30 s, followed by 40 cycles of 95 °C for 5 s and 60 °C for 30 s, ending with 95 °C for 15 s, 60 °C for 60 s, 95 °C for 15 s. The expression levels of target genes were normalized to that of glyceraldehyde-3-phosphate dehydrogenase (*GAPDH*) according to the 2^−ΔΔCT^ method [15]. The primer sequences are listed in Table 1.

### 2.6. Data Processing and Analyses

The Chroma TOF (version 4.3 ×, LECO) software was used for the analysis of peak extraction, baseline correction, deconvolution, peak integration, and peak alignment of the GC-TOF-MS data. All samples, including QC samples, were evaluated by principal component analysis model. As shown in Figures S1–S4, a high degree of aggregation of the QC samples was observed in each score plot of the liver, jejunal, ileal, and cecal content samples, indicating that the analytical performance of the instrument was stable and reproducible. The metabolites were identified qualitatively using the LECO-Fiehn Rtx5 databases. Peaks detected in less than 50% of QC samples or relative standard deviation higher than 30% in QC samples were removed. The SIMCA software package (MKS Data Analytics Solutions, Umea, Sweden) was applied for orthogonal projections to latent structure–discriminate analysis (OPLS-DA). The differential metabolites were defined as the variable importance in the projection (VIP), which was obtained by OPLS-DA > 1.0 and *p*-value < 0.05 (*p*-value was calculated from Student’s *t*-test). The relative pathways of the metabolites were searched through the Kyoto Encyclopedia of Genes and Genomes (KEGG, http://www.genome.jp/kegg/).

Results from real-time PCR analysis were presented as the mean value ± standard error of six samples per treatment (*n* = 6). The significance was determined by Student’s *t*-test for pairwise comparisons between the control and treatment groups. The difference was considered significant at *p* < 0.05.

## 3. Results

### 3.1. Overall Changes in Metabolite Profile Caused by Overfeeding

**Figure 1:**
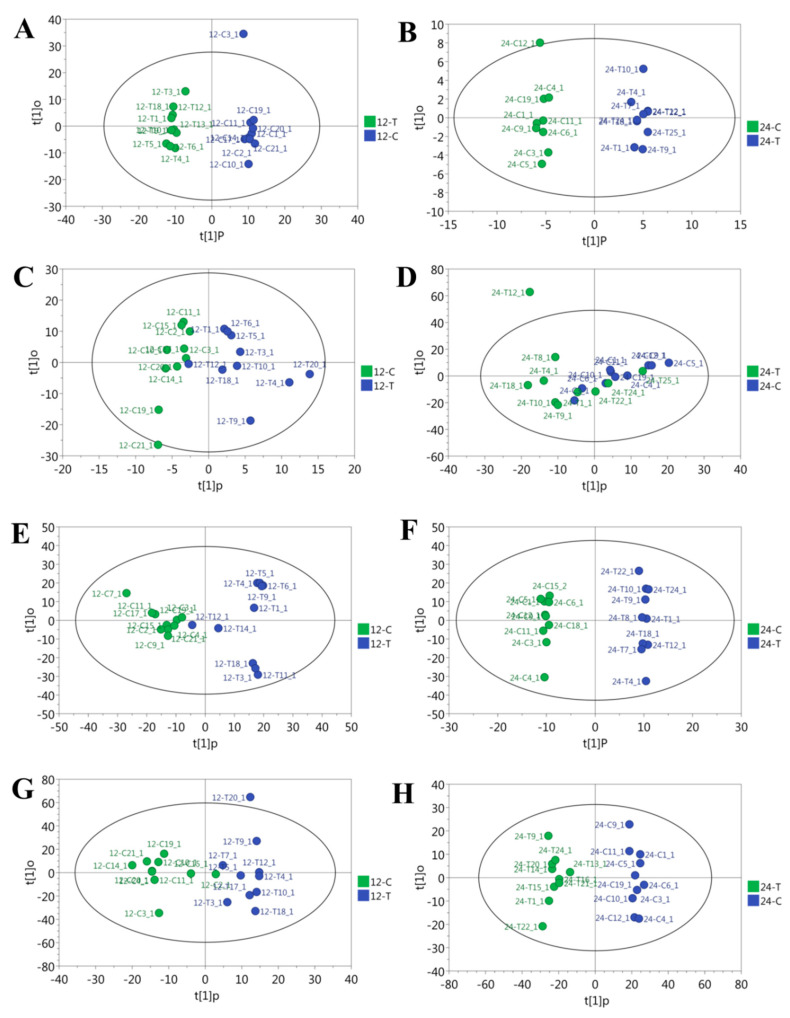
Score plot of orthogonal projections to latent structure–discriminant analyses (OPLS-DA) derived from the GC-TOF-MS profiles of samples obtained from the overfed group (T) vs. the normal control group (C) on day 12 (12-T vs. 12-C) and 24 (24-T vs. 24-C) of overfeeding. The models represent the samples of liver (**A**,**B**), jejunal (**C**,**D**), ileal (**E**,**F**), and cecal (**G**,**H**) contents, respectively.

The metabolite peaks extracted from GC-TOF-MS were 659, 530, 589, and 657 for liver and jejunal, ileal, and cecal content samples, respectively. The OPLS-DA results showed that the liver, ileal, and cecal content samples, as well as jejunal content samples, on the 12th day of overfeeding were obviously separated between the control and overfed groups (Figure 1). This suggested that overfeeding significantly changed the metabolite profiles in the liver and intestinal contents of geese.

### 3.2. Identification of Differential Metabolites

In total, 138, 28, 76, and 42 differential metabolites between the control and the overfed geese were identified in the liver, jejunal, ileal, and cecal content samples on the 12th day of overfeeding; correspondingly, 186, 11, 33, and 124 differential metabolites were identified on the 24th day of overfeeding (Table 2). These differential metabolites mainly belong to fatty acids, amino acids, organic acids, and amines. All differential metabolites and the trends of their changes are listed in Tables S1–S8.

### 3.3. Identification of Common Differential Metabolites over Different Overfeeding Times

**Figure 2:**
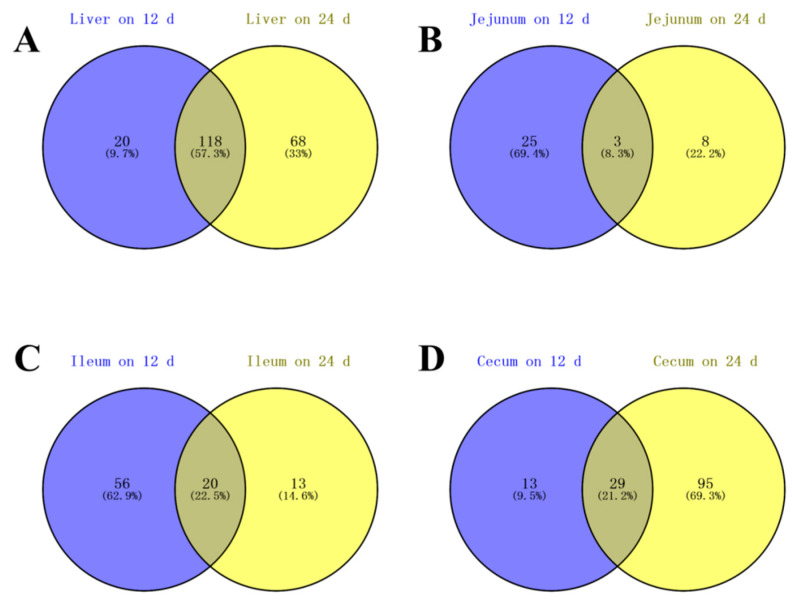
Venn diagram of differential metabolites in the liver, jejunal, ileal, and cecal contents of the overfed vs. control geese on day 12 (12 d) and day 24 (24 d) of overfeeding. The figures represent the analysis of liver (**A**), jejunal (**B**), ileal (**C**), and cecal contents (**D**), respectively.

There were 118 common differential metabolites in the liver between the 12th and 24th day of overfeeding, including 3-phosphoglycerate, ARA, ethanolamine, sphingosine, linoleic acid, 5-Dihydrocortisol, and others identified in Tables S1 and S5 (Figure 2A). In addition, there were 3 (galactonic acid, 5-dihydrocortisol, and dodecanol), 20 (24, 25-dihydrolanosterol, TD, ethanolamine, taurine, and others identified in Tables S3 and S7), and 29 (inosine, adipic acid, myo-inositol, and others identified in Tables S4 and S8) common differential metabolites identified in jejunal, ileal, and cecal content samples between the 12th day and 24th day of overfeeding, respectively (Figure 2B–D).

### 3.4. Identification of Common Differential Metabolites between the Liver and Intestinal Contents

**Figure 3:**
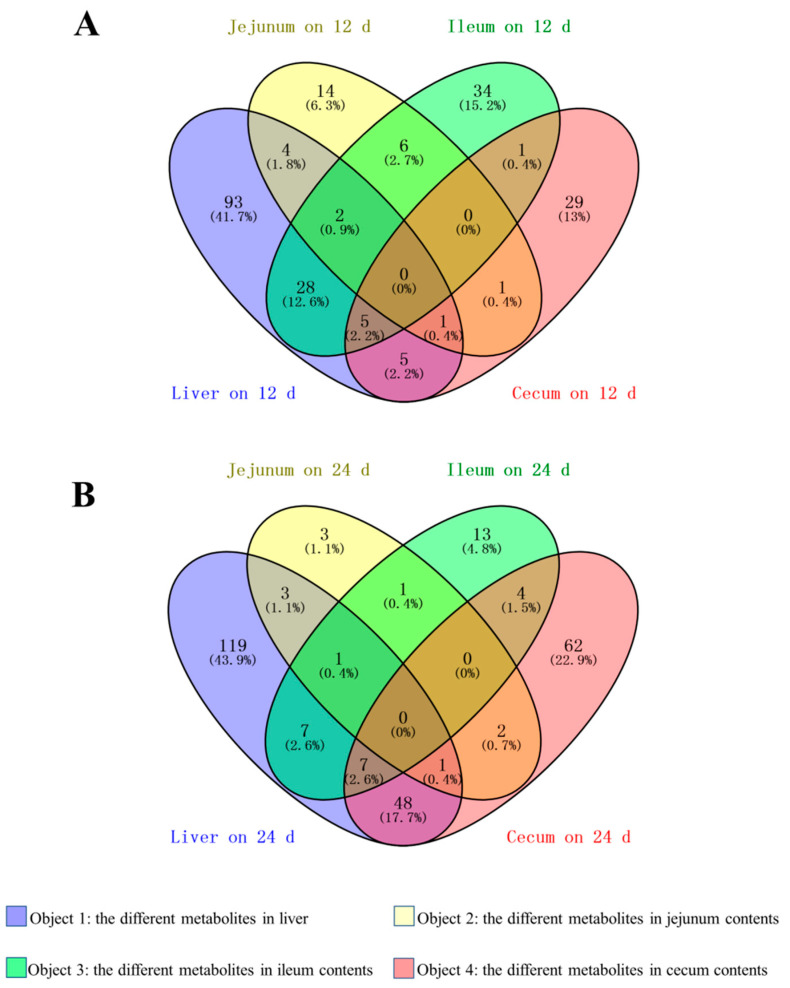
Venn diagram of differential metabolites in the liver, jejunal, ileal, and cecal contents of the overfed vs. control geese on day 12 ((**A**), 12 d) and day 24 ((**B**), 24 d) of overfeeding.

The common differential metabolites between the liver and intestinal contents are listed in Tables S9 and S10. As shown in Figure 3, there were 7 common differential metabolites between the liver and jejunal content (DL-dihydrosphingosine, 20α-Hydroxycholesterol, inosine, and others identified in Table S9), 35 common differential metabolites between the liver and ileal content (ARA, 3, 7, 12-Trihydroxycoprostane, ethanolamine, and others identified in Table S9), and 11 common differential metabolites between the liver and cecal content (inosine, L-malic acid, methyl phosphate, and others identified in Table S9) on the 12th day of overfeeding. Correspondingly, there were 5 common differential metabolites between the liver and jejunal content (nicotianamine, N-Acetyl-L-aspartic acid, galactonic acid, 5-Dihydrocortisol, O-Phosphorylethanolamine), 15 common differential metabolites between the liver and ileal content (palmitic acid, taurine, ethanolamine, 5-Dihydrocortisol, zymosterol, and others identified in Table S10), and 56 common differential metabolites between the liver and cecal content (palmitic acid, ethanolamine, linoleic acid, inosine, ARA, and others identified in Table S10) on the 24th day of overfeeding. Interestingly, it was found that short-chain fatty acids, branched-chain amino acids, and cortisol accounted for a larger proportion of the common differential metabolites between the liver and intestinal contents, suggesting that these metabolites may have a role in the formation of fatty liver.

### 3.5. The Metabolic Pathways in Which the Differential Metabolites Are Involved

As shown in Table 3, 3-phosphoglycerate and sphingosine were involved in many metabolic pathways in the liver on the 12th day of overfeeding. It is known that 3-phosphoglycerate is a key metabolite involved in glycolysis/gluconeogenesis, glycerolipid metabolism, the glucagon signaling pathway, and the pentose phosphate pathway. Sphingosine participates in sphingolipid metabolism, the sphingolipid signaling pathway, and apoptosis. Moreover, myo-inositol in jejunal content is an intermediate metabolite of multiple metabolic pathways, including ATP-binding cassette (ABC) transporters, galactose metabolism, inositol phosphate metabolism, and the phosphatidylinositol signaling system. In ileal content, taurine takes part in primary bile acid biosynthesis, ABC transporters, and taurine and hypotaurine metabolism, while ARA is involved in aldosterone synthesis and secretion, linoleic acid metabolism, the biosynthesis of unsaturated fatty acids, inflammatory mediator regulation of transient receptor potential channels, and others identified in Table 3.

On the 24th day of overfeeding, taurine was also a differential metabolite in intestinal content. Palmitic acid is a metabolite involved in the biosynthesis of unsaturated fatty acids, fatty acid biosynthesis, and fatty acid elongation (Table 4). In cecal content, L-malic acid and fumaric acid are involved in the glucagon signaling pathway, biosynthesis of terpenoids and steroids, citrate cycle (TCA cycle), pyruvate metabolism, and others identified in Table 4. In addition, cholesterol mainly takes part in primary bile acid biosynthesis, steroid hormone biosynthesis, steroid degradation, steroid biosynthesis, bile secretion, and fat digestion and absorption.

### 3.6. Effects of Arachidonic Acid and Tetrahydrocorticosterone on Gene Expression in Goose Cells

**Figure 4:**
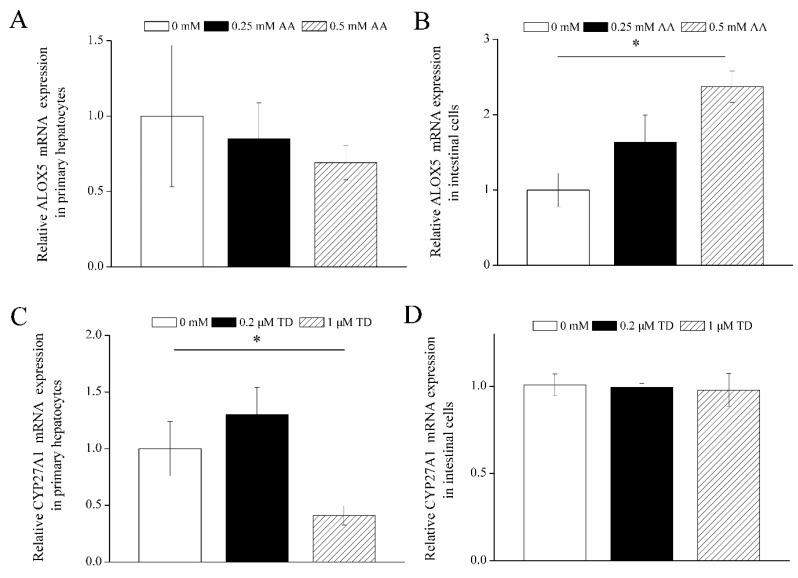
The effects of arachidonic acid (**A**,**B**) and tetrahydrocorticosterone (**C**,**D**) on the relative mRNA expression of arachidonate 5-lipoxygenase (*ALOX5*) or cytochrome P-450 27A1 (*CYP27A1*) in the primary hepatocytes and intestinal cells of goose. Primary hepatocytes and intestinal cells were isolated from goose embryos after 23 days of incubation. ARA = arachidonic acid. TD = tetrahydrocorticosterone. For ARA treatment, hepatocytes and intestinal cells that were treated with complete culture media were considered as the control group. For TD treatment, cells which were treated with complete culture media containing 2% bovine serum albumin and 0.2% alcohol were taken as the control. * *p* < 0.05. All data are presented as means ± SEM (*n* = 6).

Based on the analysis of metabolomics, ARA, sterols, and corticosteroid metabolic pathways were significantly affected by overfeeding. The data showed that the ARA content was significantly decreased in the liver of the overfed vs. control geese on the 12th day and 24th day of overfeeding (*p* < 0.05), but the ARA content in the cecum of overfed geese on 24th day of overfeeding was significantly increased (*p* < 0.05), indicating that ARA in the intestinal content may play a role in the formation of fatty liver through the gut–liver axis. The TD concentration was significantly decreased in ileal and cecal contents in the overfed vs. control geese on the 12th day and 24th day of overfeeding (*p* < 0.05), although it was not detected in the liver. In this study, primary hepatocytes and intestinal cells were treated with ARA and TD. As shown in Figure 4, compared with the control, the expression of arachidonate 5-lipoxygenase (ALOX5) in goose intestinal cells was induced by 0.50 mM ARA (*p* < 0.05); on the contrary, cytochrome P-450 27A1 (CYP27A1) RNA expression in goose primary hepatocytes was inhibited by 1 µM TD (*p* < 0.05).

## 4. Discussion

NAFLD has been considered as a considerable threat to the public health around the world. In contrast, goose fatty liver is a physiological fatty liver without obvious pathological symptoms such as inflammation or hepatic fibrosis [8,9,10]. To gain better insight into the metabolic changes that take place during the formation of goose fatty liver, we used a GC-TOF-MS method to analyze the liver and intestinal content samples in overfed vs. control geese. The OPLS-DA model indicated that there were significant differences in the liver, ileal, and cecal content samples, as well as jejunum content samples, on the 12th day of overfeeding between the control and overfed geese. These results suggest that overfeeding significantly changes the metabolic patterns in the liver and intestinal contents of geese. Moreover, there were 57.3% common differential metabolites in the liver between the 12th day and 24th day of overfeeding, but the number of differential metabolites in the cecal content was increased with overfeeding time. This suggests that more dramatic metabolic change is associated with the severity of goose fatty liver and that the metabolic change contributes to the formation of goose fatty liver.

The KEGG analysis showed that the differential metabolites in the liver were involved in pathways which are mainly associated with the metabolism of glucose, glycogen, lipid, vitamin, and sphingolipid, as well as cell apoptosis (listed in Table 3 and Table 4). It is generally believed that the formation of goose fatty liver is mainly attributed to the disruption of the balance among fatty acid synthesis, lipoprotein transport, and β-oxidation of fatty acids [8]. Glycolysis, the pentose phosphate pathway, and fatty acid synthesis are important pathways during the formation of goose fatty liver. In addition, sphingolipid metabolism and apoptosis are closely related to inflammation during the occurrence and development of NAFLD [16,17]. The results of the present study indicated that the differential metabolites in intestinal content are mainly associated with the metabolism pathways of glucose, bile acid, taurine, fatty acid, steroid hormone, amino acid, and sphingolipid (shown in Table 3 and Table 4). Moreover, many common differential metabolites between the liver and intestinal contents were observed. Taken together, these findings imply that the metabolites in jejunal, ileal, and cecal contents may affect the formation of fatty liver by influencing the metabolisms of glucose and fatty acid, oxidative stress, and inflammatory reaction.

Short-chain fatty acids, branched-chain amino acids, and sterols, especially 3-phosphoglycerate, sphingosinol, inositol, taurine, ARA, adipate, palmitic acid, and cholesterol, accounted for a large proportion of the metabolites in the liver and intestinal contents in this study. More specifically, 3-phosphoglycerate, as a key metabolite in glycolysis/gluconeogenesis, glycerolipid metabolism, the glucagon signaling pathway, and the pentose phosphate pathway, is related to carbohydrate and energy metabolism. The decrease in 3-phosphoglycerate in the fatty liver suggests that more glucose was converted to fatty acids via glycolysis in the formation of the fatty liver. Our previous study also indicates that the mRNA expression of hexokinase 1, a key enzyme of glycolysis, is significantly induced in goose fatty liver [18]. Moreover, taurine is a conditionally essential amino acid for animals. Carneiro et al. [19] reported that taurine supplementation could regulate the expression of genes required for glucose-stimulated insulin secretion, thereby increasing the insulin content in the blood and accelerating glycolysis. In addition, the other function of taurine in the liver is to combine with bile acids to form taurocholic acid, which is necessary for the absorption of lipids in the digestive tract [20,21]. The taurine level was decreased in the liver of overfed geese, suggesting that more taurine might combine with bile acids to promote lipid absorption in the intestinal tract. Previous studies found that sphingolipid metabolism was significantly changed during the occurrence and development of NAFLD, and this change was closely related to inflammation [16,17]. Sphingosine, ceramide, and sphingosine-1-phosphate can be interconverted, and their contents maintain a dynamic balance. It was reported that the concentrations of ceramide and sphingosine in the livers of obese people and animals were higher than those in the normal control group [22,23]. Ceramide can activate the NF-κB signaling pathway and promote the occurrence and development of inflammation in NAFLD [24,25]. Furthermore, ceramide and sphingosine can induce cell apoptosis, and excessive apoptosis can lead to extensive inflammatory response and necrosis in liver cells [26,27]. In the current study, the sphingosine levels were decreased in the liver and cecal content of the overfed geese. The results are consistent with our earlier findings that tumor necrosis factor-α (TNF-α) was suppressed in the goose fatty liver vs. normal liver [10]. Therefore, sphingosine may be associated with the suppression of inflammation in the goose liver, but further investigation is needed to verify the mechanism.

Oxidative stress and lipid peroxidation occur throughout the development of NAFLD [28]. Reactive oxygen species (ROS) can induce cell damage when their concentrations exceed the capacity of the antioxidant system [29]. ARA can regulate hepatic mitochondrial oxidative stress by the cyclooxygenase, lipoxygenase, and cytochrome P450 enzymes. Thus, ARA can induce oxidative stress mainly by producing a large amount of ROS, and its content may be an early indicator of inflammation and irreversible changes in NAFLD progression [30]. The results in this study showed that the contents of ARA in the liver of the overfed goose were significantly decreased, indicating that ARA may be involved in oxidative stress and inflammation in the goose fatty liver. The mRNA expression of *ALOX5*, one enzyme of lipoxygenase, was upregulated in goose primary cells, confirming that ARA can induce oxidative stress. The decrease in ARA content in the fatty liver suggests that the reduced ARA may be a protective measure against the oxidative stress and inflammation in the formation of the goose fatty liver. Glucocorticoid (GC) is one of the causes of fatty liver, as it can bind to its receptors to inhibit mitochondrial β-oxidation and induce lipid peroxidation and accumulation [31]. A large amount of evidence has indicated that glucocorticoid receptor (GR) plays an important role in the pathogenesis of NAFLD/NASH [32,33]. Jenson et al. [34] found that GR expression was decreased in the fatty liver of obese rats compared with normal individuals. Similarly, the knockout of GR in the liver induced fatty liver in mice under the state of routine diet [35]. These results suggest that the decrease in GR expression is a contributing factor in fatty liver disease. TD is a natural glucocorticoid metabolite, and its levels were decreased in the ileal and cecal contents of overfed vs. control geese. This is consistent with the above notion that the reduced binding of GC to its receptors promotes fatty liver formation. *CYP27A1* expression was inhibited in primary hepatocytes treated with TD. Gueguen et al. [36] indicated that the inhibition of *CYP27A1* expression promoted lipid deposition in tissues as it could affect the balance of cholesterol, sterol, and other lipids. We speculated that the reduced content of TD in the intestinal contents could contribute to the formation of goose fatty liver.

## 5. Conclusions

This study demonstrated that intestinal metabolites are important participants in the formation of goose fatty liver by affecting various metabolic pathways, such as the metabolisms of glucose and fatty acid, oxidative stress, and inflammatory reactions. ARA and TD may play a part in the formation of goose fatty liver by influencing oxidative stress and the lipid balance, respectively. These findings lay the foundation for addressing the relationship between intestinal metabolites and the formation of fatty liver.

## Figures and Tables

**Table 1:** Primer sequences for real-time PCR analysis.

Gene Name ^a^	GenBank Number	Primer Sequence (5′ to 3′)	Product Size (bp)
*ALOX5*	XM_013201170.1	F: CAGGGAAAGCTGGAAAACAG	240
		R: AGCTTTGCTCTTCCATCTCG	
*CYP27A1*	XM_013186353.1	F: GACCCAGCACTTCATCGACT	226
		R: CAGTGTGTTGGAGGTCGTGT	
*GAPDH*	XM_013199522.1	F:GCCATCAATGATCCCTTCAT	155
		R:CTGGGGTCACGCTCCTG	

^a^*CYP27A1*: cytochrome P-450 27A1; *ALOX5*: arachidonate 5-lipoxygenase; *GAPDH*: glyceraldehyde 3 phosphate dehydrogenase.

**Table 2:** The number of differential metabolites in the liver, jejunal, ileal, and cecal contents on day 12 and 24 of overfeeding.

Item ^a^	Liver		Jejunum		Ileum		Cecum	
	12 d	24 d	12 d	24 d	12 d	24 d	12 d	24 d
Increased	8	3	27	4	28	5	6	42
Decreased	130	183	1	7	48	28	36	82
Total	138	186	28	11	76	33	42	124

^a^ Increased or decreased represents the number of differential metabolites that has higher or lower concentration in overfed geese compared with the normally fed geese. Total means the number of total differential metabolites.

**Table 3:** Metabolic pathways identified with the different metabolites in the liver and intestinal contents between the control and overfed geese on the 12th day of overfeeding.

Different Metabolites	Related Metabolic Pathway
**Liver**	
3-Phosphoglycerate	Glycolysis/Gluconeogenesis; Glycine, serine, and threonine metabolism; Glycerolipid metabolism; Biosynthesis of terpenoids and steroids; Glucagon signaling pathway; Pentose phosphate pathway
Glutaric acid	Fatty acid degradation
3α,7α,12α-Trihydroxycoprostane	Primary bile acid biosynthesis
Sphingosine	Sphingolipid metabolism; Sphingolipid signaling pathway; Apoptosis
Lignoceric acid	Biosynthesis of unsaturated fatty acids
Squalene	Biosynthesis of terpenoids and steroids
Glutathione	ATP-binding cassette transporters; Bile secretion
**Jejunum**	
Myo-inositol	ATP-binding cassette transporters; Galactose metabolism; Ascorbate and aldarate metabolism; Inositol phosphate metabolism; Phosphatidylinositol signaling system
Raffinose	ATP-binding cassette transporters; Galactose metabolism
24, 25-Dihydrolanosterol	Steroid biosynthesis
O-Phosphorylethanolamine	Glycerophospholipid metabolism; Sphingolipid signaling pathway; Sphingolipid metabolism
**Ileum**	
3α,7α,12α-Trihydroxycoprostane	Primary bile acid biosynthesis
Taurine	ATP-binding cassette transporters; Taurine and hypotaurine metabolism; Sulfur metabolism
Arbutin	Glycolysis/Gluconeogenesis; Phosphotransferase system
Salicin	Glycolysis/Gluconeogenesis; Phosphotransferase system
Ethanolamine	Glycerophospholipid metabolism
Sucrose	Phosphotransferase system; Galactose metabolism; ATP-binding cassette transporters
Creatine	Glycine, serine, and threonine metabolism
Raffinose	ATP-binding cassette transporters; Galactose metabolism
Maltotriose	ATP-binding cassette transporters
2, 6-Diaminopimelic acid	Biosynthesis of amino acids
24, 25-Dihydrolanosterol	Steroid biosynthesis
Arachidonic acid (ARA)	Eicosanoids; gonadotropin-releasing hormone signaling pathway; Aldosterone synthesis and secretion; Linoleic acid metabolism; Biosynthesis of unsaturated fatty acids; Fc gamma R-mediated phagocytosis; Inflammatory mediator regulation of transient receptor potential channels; ARA metabolism; Regulation of lipolysis in adipocytes
**Cecum**	
Adipic acid	Degradation of aromatic compounds; Caprolactam degradation
Pipecolinic acid	Biosynthesis of alkaloids derived from ornithine, lysine, and nicotinic acid
inosine	Purine metabolism
Phenylacetic acid	Phenylalanine metabolism

**Table 4:** Metabolic pathways identified with the different metabolites in the liver and intestinal contents between the control and overfed geese on the 24th day of overfeeding.

Different Metabolites	Related Metabolic Pathway
**Liver**	
Nicotinamide	Nicotinate and nicotinamide metabolism
**Ileum**	
Glycine	Primary bile acid biosynthesis
Taurine	Primary bile acid biosynthesis; Taurine and hypotaurine metabolism; Sulfur metabolism; ATP-binding cassette transporters
24, 25-Dihydrolanosterol	Steroid biosynthesis
Zymosterol	Steroid biosynthesis
Ethanolamine	Phosphote and phosphite metabolism; Glycerophospholipid metabolism
Palmitic acid	Biosynthesis of unsaturated fatty acids; Fatty acid biosynthesis; Fatty acid metabolism; Fatty acid degradation; Fatty acid elongation
**Cecum**	
L-Malic acid	Biosynthesis of alkaloids derived from ornithine, lysine, and nicotinic acid; Glucagon signaling pathway; Biosynthesis of alkaloids derived from histidine and purine; Biosynthesis of alkaloids derived from shikimate pathway; Biosynthesis of terpenoids and steroids; Citrate cycle (TCA cycle); Pyruvate metabolism; Biosynthesis of phenylpropanoids; Biosynthesis of alkaloids derived from terpenoid and polyketide; Glyoxylate and dicarboxylate metabolism
Pipecolinic acid	Biosynthesis of alkaloids derived from ornithine, lysine, and nicotinic acid
Fumaric acid	Biosynthesis of alkaloids derived from ornithine, lysine, and nicotinic acid; Glucagon signaling pathway; Biosynthesis of alkaloids derived from histidine and purine; Biosynthesis of alkaloids derived from shikimate pathway; Biosynthesis of terpenoids and steroids; TCA cycle; Pyruvate metabolism; Biosynthesis of phenylpropanoids; Biosynthesis of alkaloids derived from terpenoid and polyketide; Phenylalanine metabolism; Nicotite and nicotimide metabolism; Arginine biosynthesis; Oxidative phosphorylation; Tyrosine metabolism; Alanine, aspartate and glutamate metabolism; Styrene degradation
Cholesterol	Biosynthesis of secondary metabolites; Primary bile acid biosynthesis; Steroid hormone biosynthesis; Biosynthesis of alkaloids derived from terpenoid and polyketide; Steroid degradation; Steroid biosynthesis; Vitamin digestion and absorption; Bile secretion; Fat digestion and absorption
Adipic acid	Caprolactam degradation; Degradation of aromatic compounds
Malonic acid	Pyrimidine metabolism; beta-alanine metabolism
Uracil	Pyrimidine metabolism; beta-alanine metabolism; Pantothete and CoA biosynthesis
Myo-inositol	ATP-binding cassette transporters; Galactose metabolism; Phosphatidylinositol signaling system; Inositol phosphate metabolism; Ascorbate and aldarate metabolism
2-Amino-3-hydroxybutyric acid	ATP-binding cassette transporters; Glycine, serine, and threonine metabolism
Glycine	Primary bile acid biosynthesis
Creatine	Glycine, serine and threonine metabolism; Arginine and proline metabolism
Ethanolamine	Glycerophospholipid metabolism; Phosphote and phosphite metabolism
O-Phosphorylethanolamine	Glycerophospholipid metabolism; Sphingolipid metabolism; Sphingolipid signaling pathway
Behenic acid	Biosynthesis of unsaturated fatty acids
ARA	Biosynthesis of unsaturated fatty acids; ARA metabolism; Eicosanoids; Linoleic acid metabolism; Aldosterone synthesis and secretion; Regulation of lipolysis in adipocytes
Lignoceric acid	Biosynthesis of unsaturated fatty acids
Tetrahydrocorticosterone	Steroid hormone biosynthesis
Inosine	Purine metabolism
24, 25-Dihydrolanosterol	Steroid biosynthesis

## Supplementary Materials

The following are available online at https://www.mdpi.com/2076-2615/10/12/2375/s1, Figure S1: Score plot of principal component analysis model derived from the GC-TOF-MS profiles of liver samples, Figure S2: Score plot of principal component analysis model derived from the GC-TOF-MS profiles of jejunal content samples, Figure S3: Score plot of principal component analysis model derived from the GC-TOF-MS profiles of ileal content samples, Figure S4: Score plot of principal component analysis model derived from the GC-TOF-MS profiles of cecal content samples, Table S1: The differential metabolites in liver on day 12 of overfeeding, Table S2: The differential metabolites in jejunum on day 12 of overfeeding, Table S3: The differential metabolites in ileum on day 12 of overfeeding, Table S4: The differential metabolites in cecum on day 12 of overfeeding, Table S5: The differential metabolites in liver on day 24 of overfeeding, Table S6: The differential metabolites in jejunum on day 24 of overfeeding, Table S7: The differential metabolites in ileum on day 24 of overfeeding, Table S8: The differential metabolites in cecum on day 24 of overfeeding, Table S9: The common differential metabolites in liver and intestinal contents on day 12 of overfeeding, Table S10: The common differential metabolites in liver and intestinal contents on day 24 of overfeeding.
