# Supplementary material for: GC-TOF-MS-Based Metabolomics Analyses of Liver and Intestinal Contents in the Overfed vs. Normally-Fed Geese
Source: Animals (Basel). 2020 Dec 11;10(12):2375. doi: 10.3390/ani10122375 (PMC7763799; doi:10.3390/ani10122375)
Supplement: Supplementary file 1 [file animals-10-02375-s001.pdf]

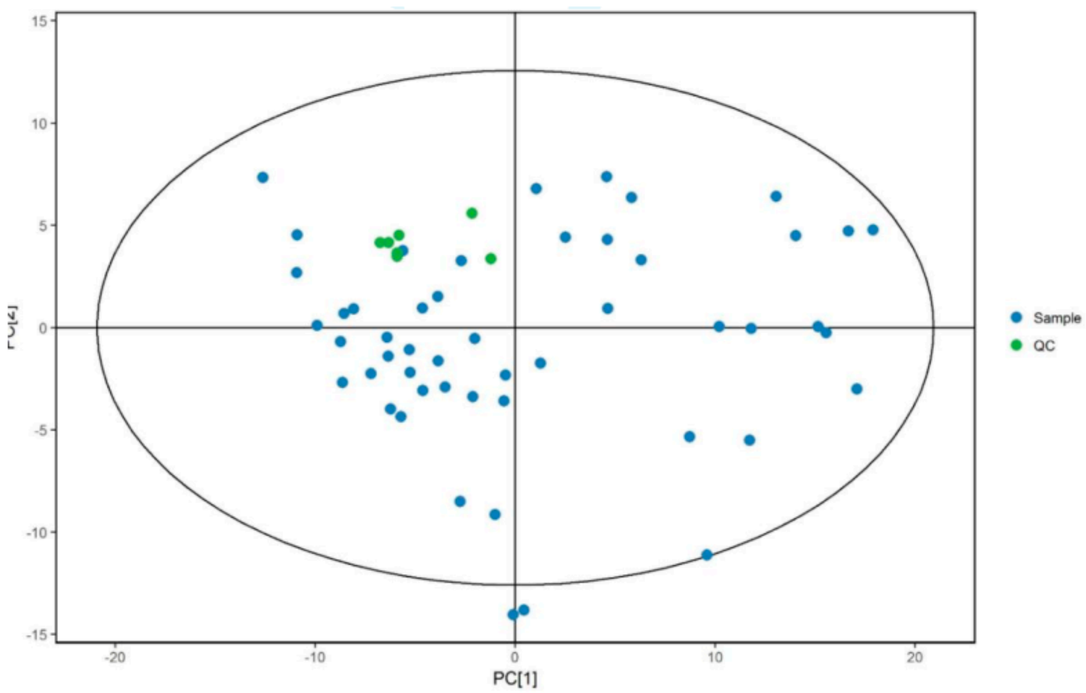

Figure S1: Score plot of principal component analysis model derived from the GC-TOF-MS profiles of liver samples. Same volume of supernatant from each sample after preprocessing was mixed as a quality control (QC) sample. Blue points represent normal samples and green points mean QC samples.

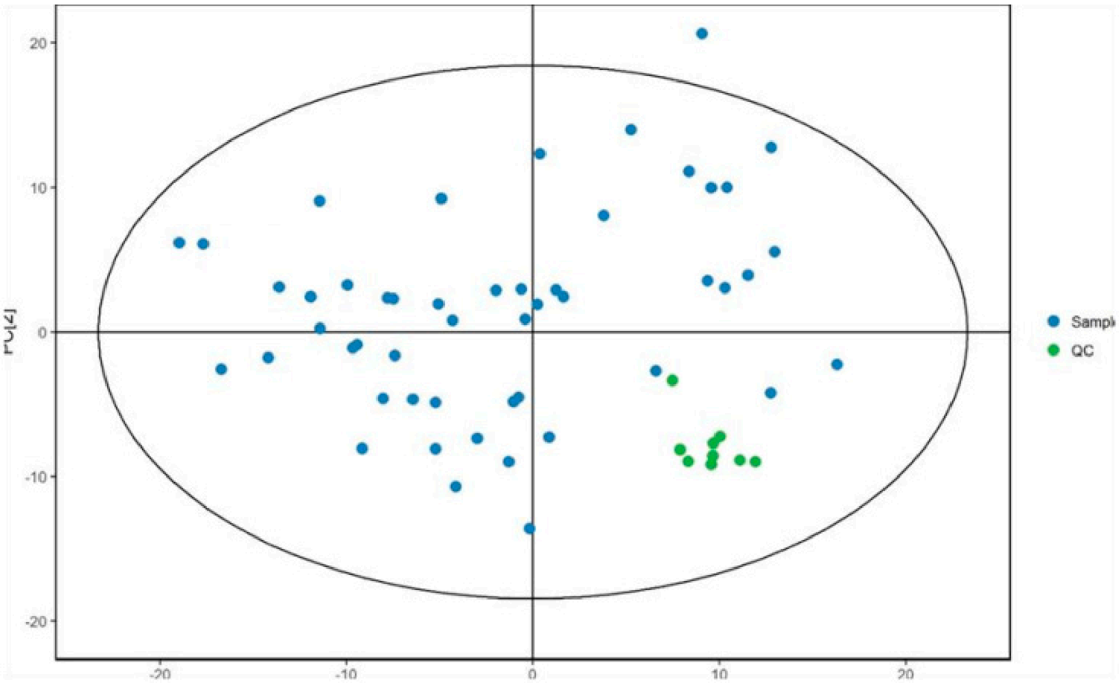

Figure S2: Score plot of principal component analysis model derived from the GC-TOF-MS profiles of jejunal content samples. Same volume of supernatant from each sample after preprocessing was mixed as a quality control (QC) sample. Blue points represent normal samples and green points mean QC samples.

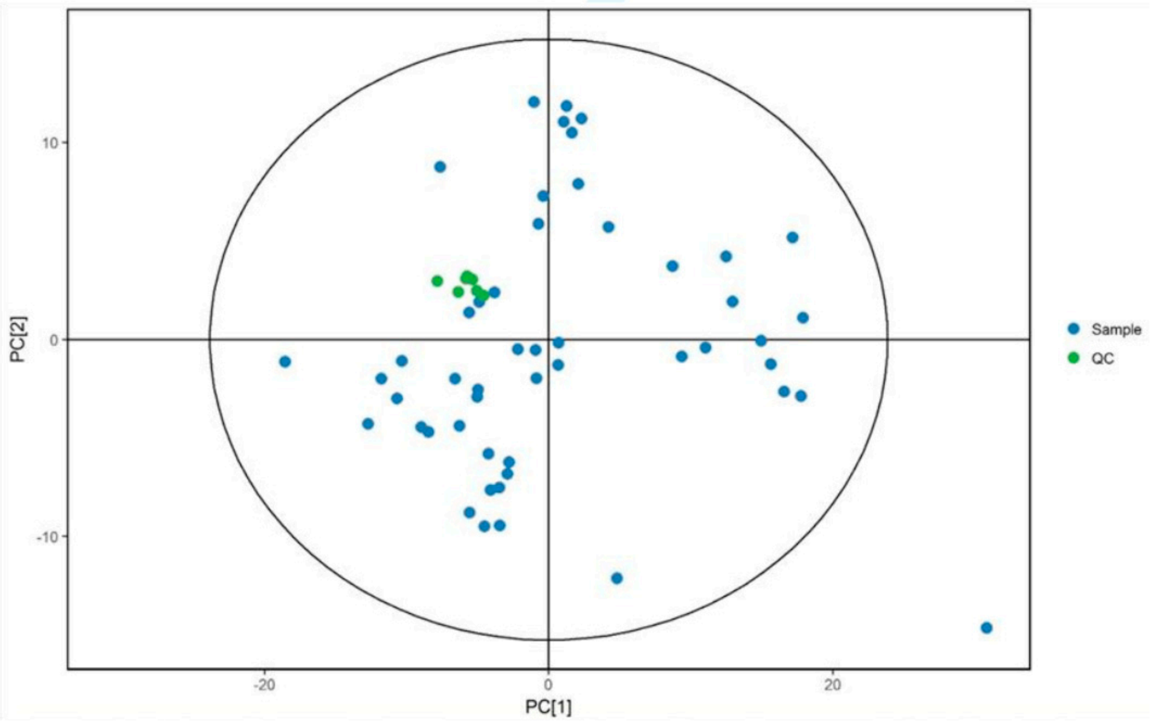

Figure S3: Score plot of principal component analysis model derived from the GC-TOF-MS profiles of ileal content samples. Same volume of supernatant from each sample after preprocessing was mixed as a quality control (QC) sample. Blue points represent normal samples and green points mean QC samples.

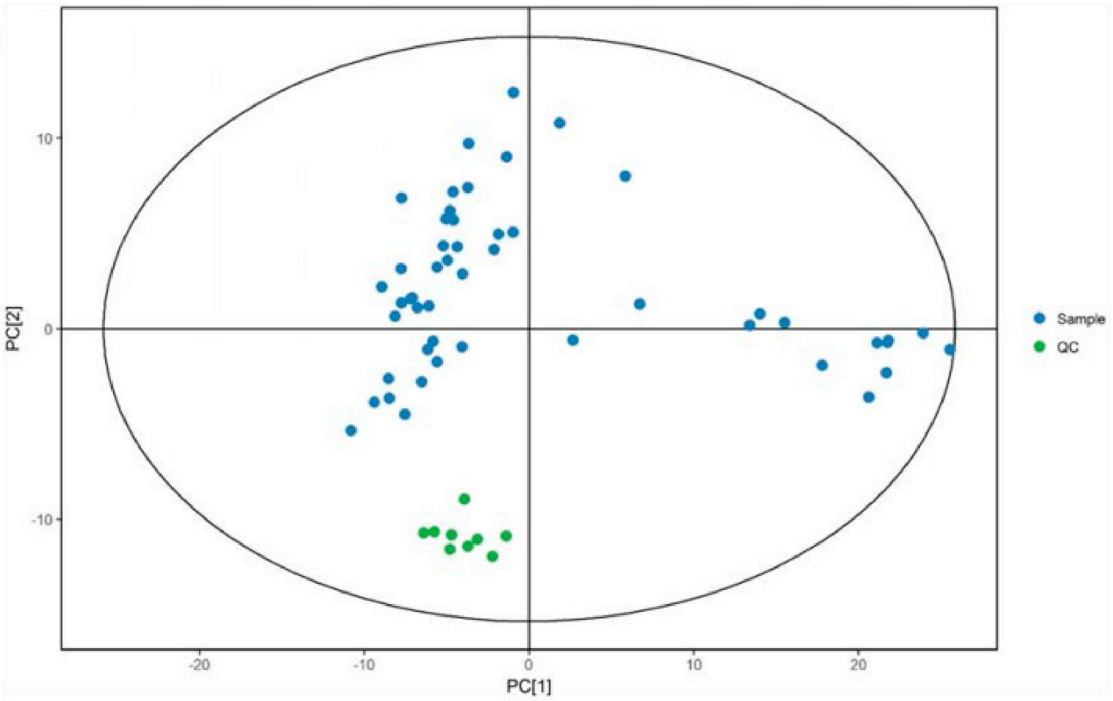

Figure S4: Score plot of principal component analysis model derived from the GC-TOF-MS profiles of cecal content samples. Same volume of supernatant from each sample after preprocessing was mixed as a quality control (QC) sample. Blue points represent normal samples and green points mean QC samples.

Table S1: The differential metabolites in liver on day 12 of overfeeding

**Differential Metabolites[1]**	**T/C[2]**	***P***	**Trend[3]**
proline	3.80	<0.001	↑
Isomaltose	0.42	<0.001	↓
guanosine	0.22	<0.001	↓
inosine	0.29	<0.001	↓
6-phosphogluconic acid	0.36	<0.001	↓
N-Methyl-L-glutamic acid	0.31	<0.001	↓
3-phosphoglycerate	0.25	<0.001	↓
5'-methylthiodenosine	0.31	<0.001	↓
L-cysteine	0.44	<0.001	↓
asparagine 4	0.18	<0.001	↓
ribose	0.30	<0.001	↓
Nicotianamine	0.28	<0.001	↓
phenylalanine	0.38	<0.001	↓
3-methylcatechol	0.42	<0.001	↓
aspartic acid	0.25	<0.001	↓
toluenesulfonic acid	0.27	<0.001	↓
arachidonic acid	0.27	<0.001	↓
5-Dihydrocortisol	0.25	<0.001	↓
fumaric acid	0.40	<0.001	↓
squalene	3.97	<0.001	↑
nicotinamide	0.16	<0.001	↓
Neohesperidin	0.30	<0.001	↓
gluconic acid 1	0.35	<0.001	↓
Cysteinylglycine	0.37	<0.001	↓
Galactinol	0.42	<0.001	↓
malonic acid	0.40	<0.001	↓
N(ε)-Trimethyllysine	0.46	<0.001	↓
Gentiobiose	0.22	<0.001	↓
tyrosine	0.51	<0.001	↓
Phenylphosphoric acid	0.56	<0.001	↓
2-ketobutyric acid	0.43	<0.001	↓
Purine riboside	0.45	<0.001	↓
Glutaric Acid	0.19	<0.001	↓
Ribonic acid, gamma-lactone	0.30	<0.001	↓
cytidine-monophosphate degr prod	0.47	<0.001	↓
oxamic acid	0.35	<0.001	↓
canavanine degr prod	0.35	<0.001	↓
2-Monopalmitin	0.37	0.001	↓
ornithine	0.19	0.001	↓
lysine	0.38	0.001	↓
oxamide	0.35	0.001	↓
prunin	0.15	0.001	↓
glutamine	0.07	0.001	↓
beta-Alanine	0.41	0.001	↓

sulfuric acid	0.63	0.001	↓
pantothenic acid	0.46	0.001	↓
D-erythronolactone	0.52	0.001	↓
xylitol	0.47	0.001	↓
sorbitol	0.39	0.001	↓
valine	0.59	0.001	↓
palmitic acid	0.68	0.001	↓
Glycine-d5	0.69	0.001	↓
linoleic acid	0.61	0.001	↓
Tartronic acid	0.31	0.001	↓
Sucrose-6-Phosphate	0.45	0.001	↓
Methyl Phosphate	0.54	0.001	↓
N-Methyl-DL-alanine	0.28	0.001	↓
cystine	0.46	0.001	↓
glutathione	0.41	0.002	↓
N-Ethylglycine	0.25	0.002	↓
2-Deoxyerythritol	0.55	0.002	↓
O-Phosphorylethanolamine	0.45	0.002	↓
Abietic Acid	0.52	0.002	↓
O-Succinylhomoserine	0.38	0.002	↓
Lactobionic Acid	0.55	0.002	↓
2,6-Diaminopimelic acid	0.46	0.002	↓
5-Methoxytryptamine	0.25	0.002	↓
resveratrol	0.41	0.002	↓
conduritol b epoxide	0.64	0.002	↓
Monostearin	0.64	0.002	↓
N-Carbamylglutamate	0.41	0.002	↓
maltose	0.49	0.002	↓
hypoxanthine	0.33	0.003	↓
stearic acid	0.66	0.003	↓
2-Methoxyestrone	0.28	0.003	↓
adenosine	0.56	0.003	↓
oxoproline	0.60	0.003	↓
3-hydroxybutyric acid	0.19	0.004	↓
sucrose	0.53	0.004	↓
uracil	0.46	0.004	↓
glutamic acid	0.44	0.004	↓
fructose-6-phosphate	0.60	0.005	↓
hexadecane	0.61	0.005	↓
21-hydroxypregnenolone	0.43	0.005	↓
succinic acid	0.67	0.006	↓
spermidine	0.56	0.006	↓
3α,7α,12α-Trihydroxycoprostane	0.66	0.006	↓
d-Glucoheptose	0.54	0.007	↓
beta-Mannosylglycerate	0.52	0.008	↓
Aminomalonic acid	0.55	0.008	↓

Ethanolamine	0.54	0.008	↓
N-cyclohexylformamide	1.29	0.008	↑
Biuret	0.40	0.009	↓
N-Acetyl-beta-D-mannosamine	0.46	0.009	↓
lactulose	0.14	0.009	↓
Dihydroxyacetone	0.43	0.010	↓
N-Acetyl-L-aspartic acid	0.46	0.010	↓
Sphingosine	0.45	0.011	↓
3,6-Anhydro-D-galactose	0.47	0.011	↓
Octadecanol	1.42	0.012	↑
DL-Anabasine	0.11	0.012	↓
Carbobenzyloxy-L-leucine degr	0.49	0.012	↓
androsterone	0.44	0.013	↓
N-Oleoyldopamine	0.34	0.014	↓
Pyruvic acid	0.30	0.014	↓
Norleucine	1.22	0.014	↑
N-Acetyl-5-hydroxytryptamine	0.33	0.015	↓
N-Acetyltryptophan	0.70	0.017	↓
2-methylfumarate	0.69	0.017	↓
3,4-Dihydroxypyridine	0.62	0.018	↓
shikimic acid	0.27	0.023	↓
xylose	0.60	0.024	↓
3-Hydroxypyridine	0.72	0.024	↓
Lactamide	0.73	0.025	↓
salicin	0.62	0.026	↓
Tagatose	0.19	0.026	↓
L-Malic acid	0.58	0.026	↓
(-)-Dihydrocarveo	0.47	0.026	↓
DL-dihydrosphingosine	0.38	0.027	↓
Isoleucine	0.74	0.027	↓
1,4-Cyclohexanedione	1.36	0.030	↑
Bis(2-hydroxypropyl)amine	0.24	0.031	↓
Lignoceric acid	0.21	0.032	↓
Fructose 2, 6-biphosphate degr prod	0.73	0.033	↓
beta-Glutamic acid	1.45	0.033	↑
Cytosin	0.60	0.034	↓
4-Vinylphenol dimer	0.62	0.034	↓
2-deoxy-D-glucose	0.63	0.034	↓
D-Arabitol	0.71	0.035	↓
3-Aminoisobutyric acid	0.75	0.037	↓
D-Glyceric acid	0.38	0.038	↓
thymine	0.40	0.038	↓
oleic acid	0.72	0.040	↓
Erythrose 2	0.63	0.042	↓
uridine	0.71	0.042	↓
threonine	1.31	0.044	↑

20α-Hydroxycholesterol	0.48	0.045	↓
glycolic acid	0.82	0.047	↓

[1]The differential metabolites were defined as the variable importance in the projection (VIP) which obtained from orthogonal projections to latent structure–discriminate analysis (OPLS-DA) > 1.0 and *P*-Value < 0.05. *P*-Value was calculated from Student's t-test.

[2]T means overfed treatment and C means control treatment.

[3]The upward and downward arrows indicate up and down trends of the metabolites in overfed treatment compared with the control treatment.

Table S2: The differential metabolites in jejunum on day 12 of overfeeding

**Differential Metabolites[1]**	**T/C[2]**	***P***	**Trend[3]**
Galactonic acid	3.02	<0.001	↑
putrescine	3.07	0.001	↑
Aldosterone	2.72	0.003	↑
Maleamate	1.77	0.006	↑
20α-Hydroxycholesterol	2.67	0.007	↑
24,25-dihydrolanosterol	1.90	0.007	↑
6-deoxy-D-glucose	1.63	0.010	↑
allose	3.16	0.010	↑
5-Dihydrocortisol	0.36	0.011	↓
myo-inositol	1.90	0.018	↑
Galactinol	4.01	0.022	↑
arbutin	3.41	0.023	↑
D-(glycerol 1-phosphate)	1.77	0.023	↑
sucrose	4.45	0.024	↑
Dodecanol	1.69	0.027	↑
inosine	1.80	0.027	↑
raffinose	3.78	0.027	↑
lactose	68.24	0.028	↑
fructose	2.36	0.029	↑
DL-dihydrosphingosine	2.19	0.030	↑
3-Hydroxypropionic acid	1.88	0.037	↑
melibiose	3.94	0.039	↑
Digitoxose	1.37	0.040	↑
conduritol b epoxide	5.45	0.042	↑
2-Amino-1-phenylethanol	1.73	0.045	↑
1-Hexadecanol	1.84	0.045	↑
Diglycerol	2.89	0.047	↑
glycocyamine	2.57	0.049	↑

[1]The differential metabolites were defined as the variable importance in the projection (VIP) which obtained from orthogonal projections to latent structure–discriminate analysis (OPLS-DA) > 1.0 and *P*-Value < 0.05. *P*-Value was calculated from Student's t-test.

[2]T means overfed treatment and C means control treatment.

[3]The upward and downward arrows indicate up and down trends of the metabolites in overfed treatment compared with the control treatment.

Table S3: The differential metabolites in ileum on day 12 of overfeeding

**Differential Metabolites[1]**	**T/C[2]**	***P***	**Trend[3]**
aspartic acid	0.20	<0.001	↓
glycine 2	0.46	<0.001	↓
lysine	0.45	<0.001	↓
glutamic acid	0.22	<0.001	↓
palatinitol	0.03	<0.001	↓
maltotriose	18.95	<0.001	↑
Dioctyl phthalate	1.24	<0.001	↑
thymine	0.45	<0.001	↓
5-Dihydrocortisol	0.11	<0.001	↓
valine	0.61	<0.001	↓
citrulline	0.42	<0.001	↓
3-Methylglutaric Acid	0.32	<0.001	↓
ornithine	0.34	<0.001	↓
threonine	0.63	<0.001	↓
arbutin	5.40	<0.001	↑
beta-Alanine	0.28	<0.001	↓
asparagine	0.42	0.001	↓
raffinose	9.06	0.001	↑
Glucose-1-phosphate	7.54	0.001	↑
24, 25-dihydrolanosterol	2.05	0.001	↑
tyrosine	0.57	0.001	↓
Atrazine-2-hydroxy	6.70	0.001	↑
creatine	0.38	0.001	↓
Sphingosine	0.12	0.001	↓
serine	0.43	0.001	↓
quinic acid	4.62	0.001	↑
Ribonic acid, gamma-lactone	4.80	0.001	↑
N-Ethylglycine	1.73	0.001	↑
2,4-diaminobutyric acid	0.24	0.002	↓
Isoleucine	0.65	0.002	↓
Ethanolamine	0.27	0.002	↓
methionine	0.38	0.002	↓
2,6-Diaminopimelic acid	0.36	0.002	↓
hydroxyurea	0.20	0.003	↓
Melezitose	12.19	0.003	↑
Gentiobiose	11.34	0.003	↑
ornithine	0.33	0.003	↓
d-Glucoheptose	0.30	0.004	↓
3,7,12-Trihydroxycoprostane	0.24	0.004	↓
1,2,4-Benzenetriol	0.53	0.005	↓
D-Talose	5.53	0.006	↑
mucic acid	1.46	0.006	↑
ribose	0.23	0.007	↓
N-Methyl-DL-alanine	0.31	0.008	↓

oxoproline	0.55	0.008	↓
alanine	0.67	0.009	↓
N-Acetyl-L-phenylalanine	5.44	0.010	↑
arachidonic acid	0.08	0.010	↓
Purine riboside	6.05	0.013	↓
taurine	0.28	0.013	↓
sucrose	29.08	0.013	↑
21-hydroxypregnenolone	0.05	0.013	↓
fumaric acid	0.36	0.013	↓
salicin	3.26	0.013	↑
Behenic acid	1.92	0.015	↑
malonic acid	0.56	0.016	↓
cholesterol	0.21	0.017	↓
N-Carbamylglutamate	0.43	0.018	↓
Tetrahydrocorticosterone	0.42	0.020	↓
fructose	3.70	0.021	↑
uracil	0.31	0.022	↓
6-methylprevitamin D	1.80	0.026	↑
lactose	3.34	0.028	↑
Abietic Acid	0.25	0.030	↓
Methyl Palmitoleate	1.99	0.031	↑
N-Acetyl-D-galactosamine	0.43	0.032	↓
allose	2.54	0.034	↑
Pyrrole-2-Carboxylic Acid	0.65	0.035	↓
Monostearin	0.29	0.036	↓
tryptophan	0.07	0.040	↓
beta-hydroxypyruvate	0.28	0.041	↓
6-deoxy-D-glucose	1.52	0.042	↑
Citramalic acid	0.30	0.043	↓
Threitol	2.10	0.044	↑
squalene	1.43	0.048	↑
Lyxonic acid, 1, 4-lactone	2.95	0.050	↑

[1]The differential metabolites were defined as the variable importance in the projection (VIP) which obtained from orthogonal projections to latent structure–discriminate analysis (OPLS-DA) > 1.0 and *P*-Value < 0.05. *P*-Value was calculated from Student's t-test.

[2]T means overfed treatment and C means control treatment.

[3]The upward and downward arrows indicate up and down trends of the metabolites in overfed treatment compared with the control treatment.

Table S4: The differential metabolites in cecum on day 12 of overfeeding

**Differential Metabolites**[1]	**T/C**[2]	***P***	**Trend**[3]
inosine	0.36	<0.001	↓
α-D-glucosamine 1-phosphate	0.23	<0.001	↓
azelaic acid	0.23	<0.001	↓
Acetol	0.17	<0.001	↓
5-Methoxytryptamine	0.25	<0.001	↓
Phenylacetic acid	0.23	0.001	↓
adipic acid	0.16	0.001	↓
Digalacturonic acid	0.27	0.002	↓
6-hydroxy caproic acid dimer	0.26	0.002	↓
Zymosterol	0.13	0.002	↓
6-Hydroxynicotinic acid	0.22	0.005	↓
1-Hydroxyanthraquinone	0.10	0.006	↓
Pipecolinic acid	0.47	0.008	↓
3-hydroxybenzoic acid	0.12	0.008	↓
beta-Glycerophosphoric acid	0.66	0.009	↓
pimelic acid	0.17	0.009	↓
ribose	0.34	0.01	↓
Glucoheptonic acid	0.46	0.01	↓
thymidine	0.59	0.011	↓
d-Glucoheptose	0.54	0.013	↓
L-Malic acid	0.58	0.014	↓
oxoproline	0.58	0.016	↓
myo-inositol	2.53	0.019	↑
pantothenic acid	0.60	0.019	↓
2,4,6-Trihydroxybenzophenone	0.22	0.019	↓
naringenin	0.22	0.02	↓
5-Aminoimidazole-4-carboxamide	0.38	0.021	↓
hydrocinnamic acid	2.17	0.021	↑
nornicotine	0.67	0.023	↓
Methyl Phosphate	2.04	0.023	↑
3-hydroxybutyric acid	0.42	0.024	↓
lactic acid	2.44	*0.03*	↑
thymine	0.70	0.031	↓
palmitoleic acid	1.25	0.035	↑
L-glutamic acid	0.50	0.038	↓
5-Aminovaleric acid	0.63	0.038	↓
benzyl alcohol	0.62	0.042	↓
Nicotinoylglycine	0.24	0.043	↓
Aminomalonic acid	2.49	0.045	↑
Dioctyl phthalate	0.35	0.047	↓
3-Hexenedioic acid	0.41	0.047	↓
glutamic acid	0.60	0.049	↓

[1]The differential metabolites were defined as the variable importance in the projection (VIP) which obtained from orthogonal projections to latent structure–discriminate analysis (OPLS-DA) > 1.0 and *P*-

Value < 0.05. *P*-Value was calculated from Student's t-test.

[2]T means overfed treatment and C means control treatment.

[3]The upward and downward arrows indicate up and down trends of the metabolites in overfed treatment compared with the control treatment.

Table S5: The differential metabolites in liver on day 24 of overfeeding

**Differential Metabolites**[1]	**T/C**[2]	***P***	**Trend**[3]
Fructose 2, 6-biphosphate degr prod	0.33	<0.001	↓
Nicotianamine	0.12	<0.001	↓
d-Glucoheptose	0.14	<0.001	↓
sulfuric acid	0.39	<0.001	↓
linoleic acid	0.20	<0.001	↓
stearic acid	0.41	<0.001	↓
Methyl Phosphate	0.23	<0.001	↓
inosine	0.12	<0.001	↓
2-ketobutyric acid	0.30	<0.001	↓
adenosine	0.21	<0.001	↓
L-Malic acid	0.07	<0.001	↓
conduritol b epoxide	0.26	<0.001	↓
Purine riboside	0.17	<0.001	↓
tyrosine	0.29	<0.001	↓
5-Hydroxyindole-3-acetic acid	0.27	<0.001	↓
arachidonic acid	0.36	<0.001	↓
6-phosphogluconic acid	0.35	<0.001	↓
androsterone	0.17	<0.001	↓
2-Deoxyerythritol	0.28	<0.001	↓
glycolic acid	0.38	<0.001	↓
Isomaltose	0.20	<0.001	↓
N-Acetyl-beta-D-mannosamine	0.14	<0.001	↓
salicin	0.23	<0.001	↓
sucrose	0.42	<0.001	↓
allose	0.21	<0.001	↓
2-Monopalmitin	0.28	<0.001	↓
xylitol	0.25	<0.001	↓
phenylalanine	0.27	<0.001	↓
spermidine	0.29	<0.001	↓
2-ketoadipate	0.72	<0.001	↓
Neohesperidin	0.22	<0.001	↓
malonic acid	0.35	<0.001	↓
cholecalciferol	0.25	<0.001	↓
guanosine	0.11	<0.001	↓
Maleamate	0.39	<0.001	↓
Acetol	0.61	<0.001	↓
Phenylphosphoric acid	0.31	<0.001	↓
Tagatose	0.10	<0.001	↓
oxamide	0.34	<0.001	↓
palmitic acid	0.42	<0.001	↓
shikimic acid	0.14	<0.001	↓
5-Dihydrocortisol	0.19	<0.001	↓
Cerotinic acid	0.25	<0.001	↓
trans-4-hydroxy-L-proline	0.38	<0.001	↓

galactose	0.46	<0.001	↓
Isoxanthopterin	0.43	<0.001	↓
fructose-6-phosphate	0.18	<0.001	↓
beta-Mannosylglycerate	0.06	<0.001	↓
hexadecane	0.44	<0.001	↓
N-Ethylglycine	0.14	<0.001	↓
heptadecanoic acid	0.27	<0.001	↓
O-Phosphorylethanolamine	0.30	<0.001	↓
Octadecanol	0.47	<0.001	↓
N-Methyl-L-glutamic acid	0.14	<0.001	↓
Abietic Acid	0.27	<0.001	↓
oleic acid	0.45	<0.001	↓
fumaric acid	0.16	<0.001	↓
5,6-dihydrouracil	0.36	<0.001	↓
5-Aminovaleric acid	0.11	<0.001	↓
uridine	0.43	<0.001	↓
Carbobenzyloxy-L-leucine degr1	0.34	<0.001	↓
3,7,12-Trihydroxycoprostane	0.59	<0.001	↓
Galactinol	0.22	<0.001	↓
succinic acid	0.33	<0.001	↓
Linoleic acid methyl ester	0.08	<0.001	↓
Atropine	0.34	<0.001	↓
cystine	0.15	<0.001	↓
prostaglandin A2	0.43	<0.001	↓
panthenol	0.24	<0.001	↓
Lignoceric acid	0.06	<0.001	↓
phosphate	0.55	<0.001	↓
beta-Alanine	0.22	<0.001	↓
nicotinamide	0.10	<0.001	↓
5-Methoxytryptamine	0.23	<0.001	↓
4-aminobutyric acid	0.38	<0.001	↓
serine	0.54	<0.001	↓
D-erythronolactone	0.37	<0.001	↓
pyrophosphate	0.55	<0.001	↓
Threitol	0.26	<0.001	↓
3,6-Anhydro-D-galactose	0.13	<0.001	↓
Carnitine	0.73	<0.001	↓
L-dopa	0.20	<0.001	↓
Glucoheptonic acid	0.13	<0.001	↓
cytidine-monophosphate degr prod	0.19	<0.001	↓
1-Monopalmitin			
